# Treatment of unstable distal-third clavicule fractures using minimal invasive closed-loop double endobutton technique

**DOI:** 10.1186/s13018-019-1073-5

**Published:** 2019-01-31

**Authors:** Yang Ruen Zheng, Yung Chang Lu, Chung Ting Liu

**Affiliations:** Department of Orthopaedic Surgery, MacKay Memorial Hospital, No. 92, Sec. 2, Zhongshan N. Rd., Taipei City, 10449 Taiwan

**Keywords:** Lateral clavicle, Fracture, Coracoclavicular stabilization, Minimally invasive, Radiologic outcomes, Clinical outcomes, Range of motion suture button, TightRope

## Abstract

**Background:**

Clavicle fractures are common clinical problems, accounting for approximately 10% of all fractures. Neer’s type II fractures disrupt the integrity of the coracoclavicular ligament and, therefore, are inherently unstable, requiring an extended period time to achieve bone union and being associated with a high rate of non- or malunion. Restoration of the stability of the distal clavicle is an important factor to decrease the rate of non- or malunion. As such, the aim of our study was to describe our technique of indirect osteosynthesis, using a minimally invasive closed-loop double endobutton (TightRope) technique for fixation of unstable (Neer’s type II) distal clavicle factures, and to evaluate the short-term clinical outcomes.

**Methods:**

Fifteen patients with a Neer’s type II fracture of the distal clavicle were treated surgically using the TightRope fixation, between January 2016 and December 2017. Clinical and radiological results were assessed using the American Shoulder and Elbow Surgeons Shoulder Score (ASES) and the Constant score.

**Results:**

Definitive fracture healing was achieved in most of the patients. No major complication was identified over a mean follow-up of 9 months, and none of the patients required additional surgery related to the index procedure. At the last follow-up, all patients had recovered functional range of motion of the shoulder, with high Constant and ASES scores, and low pain score.

**Conclusion:**

The use of TightRope fixation provided sufficient stability to reduce the medially displaced fragment of the Neer’s type II fracture, with satisfactory radiological and clinical outcomes achieved over a mean follow-up of 9 months. Future studies on the long-term outcomes are needed.

## Background

Clavicle fractures are common injuries observed in clinical practice, with the distal third of the clavicle, including the lateral attachment sites of the coracoclavicular (CC) ligament, involved in 10–28% of cases [1, 2]. Among younger patients, motor vehicle accidents and sports injuries are the most common mechanisms of trauma to the clavicle, with low energy falls being the principal cause of fracture among elderly individuals [3]. In practice, Neer’s classification is normally used to describe the type of clavicular fractures. Neer’s type II fractures occur proximal to the CC ligament, disrupting the conoid ligament, which forms the posterior and medial fasciculus of the CC ligament, which is the main stabilizer of the clavicle. Consequently, Neer’s type II fractures are significantly displaced and unstable, with the proximal fragment being drawn upwards and backwards by the effect of the trapezius, while the weight of the arm pulls the distal fragment inferiorly [4]. Because of these factors, Neer’s type II fractures are likely to progress to non- or malunion, with a non-union rate of 25–44% for cases not managed surgically [2, 4–8]. Therefore, surgical treatment is recommended for Neer’s type II distal clavicle fractures.

Different surgical approaches have been developed for the treatment of Neer’s type II fractures, including direct osteosynthesis, using a locking plate, hook plate, or K-wire, or indirect stabilization of the CC, using various suture materials or tendon grafts for reconstruction [9–12]. More recently, arthroscopically assisted fixation techniques have also been described [13, 14]. Among these possible treatment techniques, direct osteosynthesis, such as hook plate construction, is associated with a high rate of complications, requiring a second operation for implant removal to prevent hardware-associated complications [15, 16]. Based on previous reports of excellent clinical outcome of indirect CC stabilization, we present our experience in performing a minimally invasive, closed-loop, double endobutton stabilization of Neer’s type II distal clavicle fractures, using the TightRope system (Arthrex, Naples, FL, USA), and describe the short-term clinical outcomes [4, 17].

## Methods

### Statement of ethics and description of the patient group

Our study was approved by our institution’s Committee for Research Ethics. Informed consent was obtained from all individual participants included in the study. We conducted a retrospective analysis of 15 patients who were treated for a Neer’s type II fracture of the clavicle using a minimally invasive reconstruction of the CC, between January 2016 and December 2017. All patients underwent fluoroscopy-guided indirect reduction and osteosynthesis via CC fixation, using the closed-loop double endobutton technique, through a single incision, with the TightRope™ system.

### Surgical technique

General anesthesia was administered, and the patient was placed in a beach chair position. A 4–5 cm incision was performed, extending from the coracoid process to the distal clavicle, with penetration through the deltotrapezial interval and separation of the deltotrapezial fascia from the clavicle to expose the fracture site. Inferiorly, the coracoid process was identified and the fracture site was cleared of interposed soft tissue. Fracture reduction was performed using a downward pressure on the proximal fragment, and a reduction clamp was applied to maintain the temporary reduction. Using a power drill, a 2.4-mm drill tip guide pin, inserted in a guide pin sleeve, was advanced through the proximal clavicle fragment and coracoid process. The tip of the guide pin was advanced only to the base of the coracoid process, under direct visualization, to avoid breaching of the undersurface of the coracoid. A 4-mm cannulated drill tip was then used to over-drill over the guide pin, creating a tunnel through both the clavicle and coracoid process. Subsequently, an implant guide sleeve was inserted through both tunnels, in a superior-inferior direction, and the TightRope implant was inserted into both tunnels using an implant pusher. Once the pusher was fully advanced, it was withdrawn, together with the implant guide sleeve.

Withdrawal of the pusher triggered the oval, metallic endobutton to flip into a horizontal position against the inferior surface of the coracoid process. Subsequently, the fiber wires were alternately pulled to place the endobutton flat against the distal clavicle tunnel. After satisfactory tension was achieved, knots were made on top of the round button to complete the fixation. Fluoroscopy was used to confirm the fracture reduction, followed by suturing of the acromioclavicular capsule, using 2-0 FiberWire, and standard closure of the incision site.

### Surgical technique

The postoperative protocol consisted of immobilization in a sling, with the shoulder in a position of internal rotation and abduction, for 4 weeks, with pendulum movements permitted from postoperative day 1. Full range of motion of the shoulder and strengthening exercises were initiated on postoperative week 4.

The postoperative follow-up included a physical examination and plain radiographs (posterior-anterior view), performed at 2-week interval over the first 2 months after surgery and then monthly from postoperative months 3 to 6. At each follow-up visit, patients completed the Constant and the American Shoulder and Elbow Surgeons Standardized Shoulder Assessment (ASES) of daily function. Postoperative complications were also assessed, including the need for re-operation, infection, implant failure, and fracture non-union.

In all cases, patients had recovered full range of movement of the shoulder and had returned to their normal physical activity by the follow-up at 12 weeks post-surgery.

## Results

Patient and clinical characteristics are reported in Table 1 and summarized as follows. The mean age of our study group was 49.67 ± 14.32 years (range, 32 to 72 years). The mechanisms of injury included motorcycle accidents (*n* = 11); sports injuries, resulting from lifting heavy objects (*n* = 1); and fall-related injuries (*n* = 3). The mean duration of the follow-up was 9 months (range, 7–12 months).

**Table 1:** Epidemiology and mechanism of clavicle fracture

Patient	Age (years)	Sex	Mechanism of injury	Side	Dominant arm	Surgery (days)	Admission (days)	Complications
1	58	M	MVA (motor bike)	R	R	1	1	–
2	32	M	Sports injuries	R	R	3	2	Peri-implant fracture of coracoid process
3	72	M	MVA (motor bike)	L	R	2	23	–
4	64	F	Fell down	R	R	2	2	–
5	52	M	MVA (motor bike)	R	R	1	1	–
6	64	F	Fell down	L	R	2	2	–
7	36	F	MVA (motor bike)	R	R	1	1	–
8	65	F	MVA (motor bike)	R	R	1	2	–
9	30	M	MVA (motor bike)	L	R	1	1	–
10	63	M	Fell down	L	R	1	2	–
11	39	F	MVA (motor bike)	L	R	1	1	–
12	51	M	MVA (motor bike)	R	R	1	2	–
13	50	F	MVA (motor bike)	R	R	1	2	–
14	37	F	MVA (motor bike)	R	R	1	1	–
15	32	M	MVA (motor bike)	R	R	1	1	–
AVE + SD	49.67 ± 14.32					1.33 ± 0.62	2.93 ± 5.57	–

*M* male, *F* female, *MVA* motor vehicle accident, *SD* standard deviation

### Clinical and functional outcomes

The average length of time from surgery to the last out-patient follow-up was 6 months. At the last follow-up assessment, the mean pain score (measured on a 10-point visual analog scale) was 1.40 ± 0.51, with a mean ASES score of 88.27 ± 7.93 and mean Constant score of 92.33 ± 4.89. The final functional outcome scores and motion improvement are reported in Tables 2 and 3. All patients were satisfied with the results, and most of them returned to work after the last follow-up.

**Table 2:** Clinical outcomes and range of motion at the final follow-up

Patient	ASA physical status score		Forward rotation (°)		External rotation (°)		Abduction (°)		ASES score		Constant score	
1	7	1	100	160	60	70	90	160	20	81	19	90
2	7	1	80	120	40	50	50	120	15	92	24	94
3	9	2	20	100	10	30	30	100	10	73	14	86
4	7	1	30	110	30	50	70	160	32	94	18	94
5	6	1	120	160	60	70	90	180	30	97	24	98
6	7	2	100	120	30	50	50	120	20	82	22	86
7	8	2	60	110	30	70	30	100	22	90	20	90
8	7	1	100	120	60	60	50	120	20	82	24	86
9	7	1	60	100	60	70	50	100	22	78	22	90
10	8	2	60	100	50	60	30	100	15	81	14	86
11	7	1	90	110	50	60	60	120	22	97	24	98
12	7	2	90	110	40	60	60	120	20	94	22	98
13	8	2	80	120	40	70	50	110	15	92	20	97
14	6	1	100	120	60	80	90	110	90	97	24	98
15	6	1	100	120	60	70	90	160	30	94	22	94
Mean	7.13 ± 0.83	1.40 ± 0.51	79.33 ± 28.15	118.67 ± 18.46	45.33 ± 15.52	61.33 ± 12.46	59.33 ± 22.19	125.33 ± 26.42	25.53 ± 18.84	88.27 ± 7.93	20.87 ± 3.40	92.33 ± 4.89
*P* value		1.26255E-17		6.84774E-05		0.002186871		2.73669E-08		1.729E-10		

*ASES* American Shoulder and Elbow Surgery Score

**Table 3:** Difference in the ASA, ASES, Constant score, and range of motion of patients before and after surgery

Mean (range)			
Variable	Pre-operatively	Postoperatively	*P* value
ASA physical status score	7.13 ± 0.83 (7–9)	1.40 ± 0.51 (1–2)	< 0.05
External rotation (°)	45.33 ± 15.52 (10–60)	61.33 ± 12.46 (30–70)	< 0.05
Forward rotation (°)	79.33 ± 28.15 (20–120)	118.67 ± 18.46 (100–160)	< 0.05
Abduction (°)	59.33 ± 22.19 (50–90)	125.33 ± 26.42 (100–180)	< 0.05
ASES score	25.53 ± 18.84 (10–32)	88.27 ± 7.93 (73–97)	< 0.05
Constant score	20.87 ± 3.40 (14–24)	92.33 ± 4.89 (86–98)	< 0.05

*ASES* American Shoulder and Elbow Surgery Score

### Radiographic outcomes

All patients completed all radiographic assessments. There were no failures of the fixation or loss of reduction over the follow-up period, and bony union was achieved in all cases.

### Complications

**Fig. 1:**
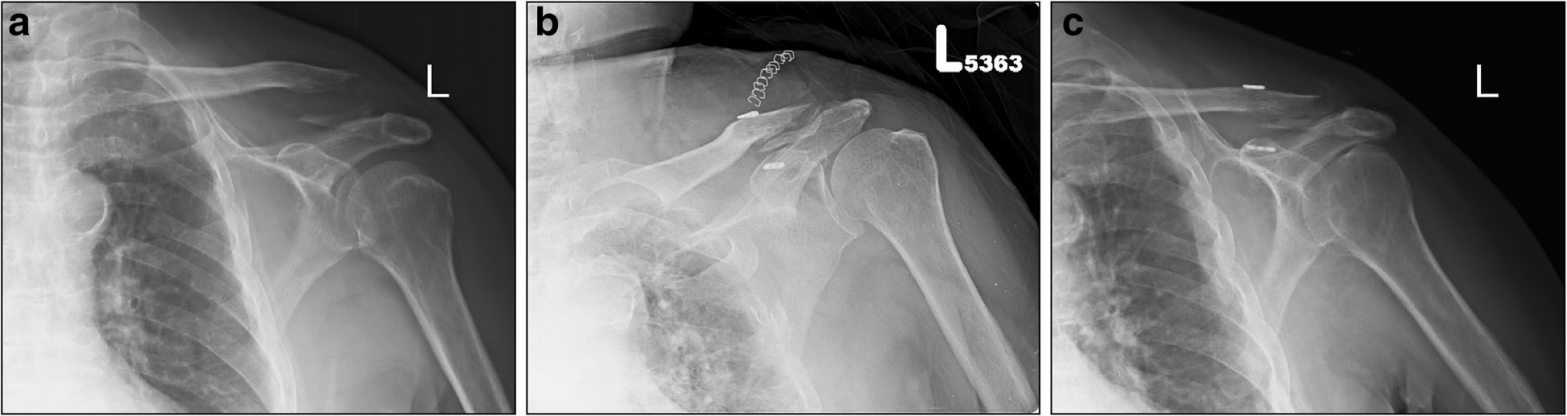
A 32-year-old male with a left distal clavicle fracture (Neer type IIA). During the steps of bone tunnel creation, we changed the trajectory of drilling through the clavicle and coracoid process (**a**). The endobutton is shown migrating through the inferior surface of the coracoid process due to an occult fracture of the base of the coracoid (**b**). Posterior-anterior plain radiograph obtained 2 weeks after surgery (**c**)

No major complications were noted, including the absence of a loss of reduction or deep infection. Only one complication was noted, a peri-implant fracture of the coracoid process, causing an upward migration of the endobutton into the coracoid process (Fig. 1a–c). Over the follow-up period, none of the patients required re-operation to remove the implant and all patients returned to their daily activities within 6 weeks and work activities, without restriction, within 3 months.

## Discussion

The definitive treatment for distal clavicle fracture remains a challenge, especially for Neer type IIB distal clavicle fractures, which are inherently unstable and associated with a high rate of non-union, ranging between 25 and 44%, with non-operative treatment [4–8, 18, 19]. With regard to the surgical treatment, a retrospective meta-analysis of 425 cases reported a complication rate of 1.6 to 22% for direct osteosynthesis [15], with a specific risk for subacromial impingement when using a hook plate fixation. To reduce the distal fragment of the fracture, the hook part of the plate is inserted below the acromion, posterior to the acromioclavicular joint, to disperse the stress of the fracture to the acromion. However, this procedure results in the osteolysis of the acromion and subacromial impingement in the majority of cases (68%), leading to the necessity for removal of the hook plate once bony union is achieved [20, 21]. In a recent study, Tan et al. reported that 74% of patients treated using a hook plate reported persisting mild-to-severe shoulder pain [22]. A small prospective study reported the development of a rotator cuff lesion, in the posterior third of the supraspinatus tendon, in 15% patients of cases, with the highest risk being in older patients with pre-existing shoulder pathology. Again, in these cases, removal of the implant after a bony union is necessary.

Stable fracture fixation could be achieved using specialized lateral locking plates with multidirectional locking screws placed into the distal clavicle fragment, avoiding secondary acromioclavicular and rotator cuff injuries [23]. Although the use of lateral clavicle locking plates has been associated with a low rate of complications and high rate of bony union, removal of the implant is required in 40% of patients due to discomfort during daily activities [24]. Moreover, the use of lateral clavicle locking plates is limited in cases in which the distal fragment in comminuted fractures of the clavicle is small, due to the difficulty of finding appropriately sized screws.

With regard to indirect flexible osteosynthesis fixation, CC stabilization and the use of the suspensory loop system are the two principal fracture stabilization techniques. In 1990, Neer published a surgical fixation technique for unstable shoulder girdle fractures using braided polyethylene sutures [25]. Largo et al. recommended the use of CC augmentation to reduce a small and/or comminuted distal clavicle bone fragment in unstable clavicle fractures to prevent high shearing forces on the proximal fragment [26]. Several case series have reported excellent rates of bony union for open reduction and internal fixation of the distal clavicle fragment using a contoured locking plate and suspensory loop system [27]. As the development of flexible osteosynthesis has progressed, excellent outcomes have been reported with the use of the suspensory loop system alone for the treatment of unstable clavicle fractures [28].

The acromioclavicular TightRope device was initially designed to stabilize ankle syndesmotic injury, with subsequent application for the stabilization of acromioclavicular joint separation. In a biomechanical study on a fresh-frozen cadaver, comparing different devices for the treatment of unstable, comminuted distal-third clavicle fractures, load to failure tension, stiffness of the fixation, and degree of fragment displacement were comparable for the locking plate and TightRope fixation methods [29]. The TightRope system can, therefore, effectively reduce the fracture fragment, converting an unstable Neer type II fracture pattern into a stable pattern, with less soft tissue dissection being required than with direct osteosynthesis, which may facilitate fracture healing especially in elderly osteoporotic patients. The relative stability techniques with flexible osteosynthesis was used to reduce the excessive strain on the osteoporotic bone. This may result in reducing the risks of microfracture, resorption of the bone, and failure of fixation.

In a recent small case series study, the use of a suture button device, including the TightRope device, was associated with good functional outcomes and radiographic results [30]. In their case series of 18 patients with a distal clavicle fracture treated using a TightRope system, Cho et al. reported a bony union rate of 94.4% (17/18 patients), with a mean ASES score of 88.6 [31].

Satisfactory clinical and functional outcomes have been reported for osteosynthesis performed using arthroscopy-assisted fixation of distal clavicle fractures. An arthroscopic approach allows for anatomic reduction of the fracture and identification and treatment of intra-articular pathology [26, 32]. In our experience, however, we have found that proper use of a fluoroscope was sufficient, with confirmation of fracture reduction, avoiding the prolonged surgical time and additional ports required for arthroscopy-assisted fixation. With regard to combining our minimally invasive approach with the TightRope device, we need to consider the findings from previous studies that have reported that this device provides only monoplanar (superior-inferior) stabilization, which can result in an anteroposterior translocation [33]. Other studies, however, reported no difference in the anterior-posterior and superior-inferior stability using the TightRope system when a modified technique, using double clavicle tunnels, was used [34]. In our experience, the monoplanar (superior-inferior) stabilization provided by the TightRope device is sufficient for a Neer type II clavicle fracture, without a risk for anterior-posterior translocation. Moreover, we only experienced one complication in our study group, due to a technical error, in which the trajectory of the drill bit through the clavicle and coracoid process resulted in an occult fracture of the base of the coracoid. Therefore, when we subsequently pulled the traction sutures to flip the endobutton onto the inferior surface of the coracoid process, the button migrated into the coracoid process at the site of the occult (Fig. 1a–c). Although no absolute contraindication of the use of the TightRope technique for the reduction of distal clavicle fractures has previously been reported, we consider that a concomitant fracture of the coracoid process is an absolute contraindication as the use of the TightRope technique in those patients may cause implant migration. Although coracoid fractures are the contraindication for TightRope devices and fractured coracoid process is easily overlooked when the focus is directed towards the clavicle fracture, preoperative radiographs of computed tomography scan to confirm the injured shoulder if it combined with the coracoid fracture are not necessary. Initial radiographs included ipsilateral and contralateral Zanca views, anteroposterior and axillary lateral views, and view of the shoulder which are useful for comparison and demonstrating the coracoids fracture.

For comminuted distal clavicle fractures with avulsion fractures of the coracoclavicular ligament (Neer type V), TightRope devices are not suitable for distal fragment reduction. The distal avulsion fragment is difficult to be reduced; thus, the distal locking plate would be a better option for bone fragment reduction and fixation. However, in our experience, the comminuted distal clavicle fracture fixed with TightRope devices resulted in acceptable functional outcome and patient satisfaction and no bone non-union occurred.

## Limitations

There are a few limitations in our study that should be acknowledged. Foremost, this was a retrospective case series study, with no randomization with another treatment technique and no control group. All surgeries were performed by one surgeon at a single site, and the sample size (*n* = 15) was small. Lastly, our evaluation was over a short-term follow-up of 6 months, with studies examining longer-term outcomes being needed.

## Conclusions

The TightRope system can provide sufficient strength to reduce the displaced medial fragment of an acute Neer type II clavicle fracture, with satisfactory radiographic and short-term clinical outcomes. The bone union rate was acceptable, with only 1 complication noted among our 15 cases due to a technical error. No clinical evidence of shoulder impingement was noted, and there was no need for re-operation for implant removal over the 6-month period of follow-up observation. Our minimally invasive technique, using the TightRope device, shortened the surgical time, length of hospital stay, and wound healing time compared to traditional techniques. The TightRope device is a promising option for fixation of unstable distal clavicle fractures.

## Abbreviations

ASES: American Shoulder and Elbow Surgeons Standardized Shoulder Assessment
CC: Coracoclavicular
